# The Sense of Belonging to the Country: Integrative Relationships and Spatiotemporal Commitment

**DOI:** 10.3389/fpsyg.2021.635113

**Published:** 2021-06-29

**Authors:** Aleksandrs Kolesovs

**Affiliations:** Department of Psychology, University of Latvia, Riga, Latvia

**Keywords:** sense of belonging, country, involvement, acceptance, commitment, temporal frame, considering emigration

## Abstract

The satisfaction of the need to belong reflects in the sense of being an integrative part of the group or social system. There is some lack of empirical evidence for the structure of this sense at the macro level. This study assessed a two-dimensional model of the sense of belonging to the country, which included relational and spatiotemporal components. Participants were 539 university students from 18 to 50 (74% females). Questions regarding involvement, perceived acceptance, sense of commonality, and feeling at home represented the relational component of the sense of belonging. Four temporal categories—the recent past, present, and the near and distant future—were included in the assessment of its spatiotemporal component. A confirmatory factor analysis revealed an acceptable fit of the two-factor model. Its convergent validity was demonstrated by the association with an explicit single-item measure of belonging. The predictive effect of the spatiotemporal component emphasized the importance of continuity of belonging in considering emigration. In sum, the results confirmed the complexity of the sense of belonging to the country and the interconnectedness of integrative relationships and spatiotemporal commitment and revealed functional differences between them.

## Introduction

A need to relate and belong to other people is among the basic human needs and motivators (Baumeister and Leary, 1995; Deci and Ryan, 2000). The satisfaction of this need results in the sense of belonging and personal involvement in a social system and physical or cultural environment (Hagerty et al., 1992; David and Bar-Tal, 2009). Gradual involvement in different social systems and a broad range of cultural and physical environments are reflected in a variety of specific senses of belonging (Miller, 2003). The sense of belonging to the country addresses the level of society. This sense constitutes part of the national identity (Phinney and Ong, 2007; Fuller-Rowell et al., 2013), and expresses in strategies of acculturation (e.g., Berry and Hou, 2016). Theoretical analyses revealed the complexity of the sense of belonging to the country (Miller, 2003; Anthias, 2011). Simultaneously, it is operationally defined as two or one factor (Dekel and Nuttman-Shwartz, 2009; Dekel and Tuval-Mashiach, 2012) or even one explicit item (e.g., Berry and Hou, 2016). This study aimed at reassessing the complex structure of the sense of belonging to the country.

A general definition of the sense of belonging refers to “the experience of personal involvement in a system or environment so that persons feel themselves to be an integral part of that system or environment” (Hagerty et al., 1992, p. 173). Simultaneously with individual involvement in relationships, perceived acceptance from others forms the basis for the sense of belonging and experiencing integration in systems and environments (Anthias, 2011; Banting and Soroka, 2012). A social-belonging intervention confirmed an increase in the sense of belonging in students, who were doubted in their acceptance (Walton and Cohen, 2011). Both—involvement and acceptance—reflect positive relationships with other people and constitute a relational component of the sense of belonging.

From a broader perspective of social identity (Phinney and Ong, 2007; Leach et al., 2008; David and Bar-Tal, 2009), the sense of belonging reflects psychological affiliation with a group at different levels of social systems and is among components of the social identity (e.g., self-categorization, exploration, values). This sense is also referred to as an affective commitment, emphasizing attachment to the group, interdependence of people (Phinney and Ong, 2007; Fuller-Rowell et al., 2013), their solidarity (Leach et al., 2008), and feeling in common with in-group members (Cameron, 2004). At the national level, the sense of belonging reflects psychological affiliation with or commitment to the country (Phinney and Ong, 2007; David and Bar-Tal, 2009). These ties promote national identification (Cameron, 2004) and solidarity (Leach et al., 2008).

The analysis of identity actualizes its temporal dimension, connecting the past, present, and future (e.g., Miller, 2003; Anthias, 2011) and forming continuity of identity (Sani et al., 2008; David and Bar-Tal, 2009). Empirically, the significance of historical ties and prospects for the sense of belonging is demonstrated at the community level (e.g., Arcidiacono et al., 2007). At the macro-level, prospective associations of personal life with the country (Dekel and Tuval-Mashiach, 2012; Kolesovs, 2019) are explored separately from a retrospective view of these ties (Shin et al., 2014). It indicates a need for an integrated analysis of the temporal dimension of the sense of belonging that includes perceived ties with the country in the past, present, and future.

This short overview demonstrates that the sense of belonging to the country can be considered a multifaceted construct, which needs further investigation. The current model proposes that the relational component of the sense of belonging to the country includes personal involvement, perceived acceptance, sense of commonality, and feeling at home (Hagerty et al., 1992; Cameron, 2004; David and Bar-Tal, 2009; Dekel and Nuttman-Shwartz, 2009; Anthias, 2011; Berry and Hou, 2016). The spatiotemporal component of belonging represents personal commitment to the country in the past, present, and future (Miller, 2003; Sani et al., 2008; Anthias, 2011; Dekel and Tuval-Mashiach, 2012; Kolesovs, 2019).

In the last two decades, Latvia has faced the problem of emigration. Its level competed with natural mortality among factors of depopulation (e.g., Central Statistical Bureau of Latvia, 2016). University students were selected for testing the model of belonging as a group actively considering various plans and opportunities, including emigration. Holmes and Burrows (2012) indicated a contribution of the sense of belonging to considering emigration and re-emigration. Simultaneously, there is some lack of quantitative assessment of this contribution, which can be performed with the suggested model.

## Method

The study was reviewed and approved by the Research Ethics Committee of the Institute of Cardiology and Regenerative Medicine of the University of Latvia, No 125/2020. The convenience sample included 500 university students from Riga, Daugavpils, and Valmiera. Participants were 18 to 49 (mean age = 24.26 years, *SD* = 6.55, 73% were females). Graduate students constituted 25% of the sample, 57% of the participants worked, 56% of the students indicated that their income was higher than the median, and 16% of the participants were married. Latvian speakers (the majority in Latvia), Russian speakers (the largest minority group), and students from other ethnolinguistic groups or bilinguals formed 79, 19, and 2% of the sample, respectively.

Test–retest reliability of measures of the sense of belonging was established in a separate sample of 39 participants. They were 20 to 50 (mean age = 31.15, *SD* = 9.41, 79% females). The retest interval varied from 3 to 4 weeks.

The spatiotemporal component of belonging to the country was assessed by using the relevant part of The Sense of Belonging in Social Context Questionnaire applied by Kolesovs (2019) for addressing commitment to Latvia. Students answered the question: “To what extent do you associate your life with Latvia?” The level of association was assessed for each of four temporal categories: the recent past, present, near future, and distant future. Participants used a seven-point scale from “minimally” (1) to “maximally” (7) to rate their answers. The distinction between the near and distant future was based on considerations regarding temporal construal (Trope and Liberman, 2003). The recent past was added to three items for an integrated view of belonging in time. The previous study (Kolesovs, 2019) revealed Cronbach’s alpha of 0.80, confirming acceptable internal consistency for three items (the present and the near and distant future).

The measure of the relational component of the sense of belonging to the country included four items: “I feel accepted in Latvia,” “I take an active part in the life of Latvia,” “I feel a commonality with the people of Latvia,” and “I feel at home anywhere in Latvia.” Students also used a seven-point scale from “completely disagree” (1) to “completely agree” (7) to rate their answers.

By analogy with Berry and Hou (2016), a single item was applied for testing a convergent validity of both subscales, measuring the sense of belonging to the country. Students posed their agreement to the following statement: “I feel belonging to Latvia.” The seven-point scale mentioned above was used for this item.

Students’ consideration of emigration was assessed within a frame of future-oriented behavior (Seginer et al., 2004), including exploratory activities and commitment followed by planning and implementing these plans. Participants rated four items using the seven-point scale, anchored by “completely disagree” and “completely agree”: “I am looking for information on emigration opportunities;” “I have chosen the country I will go to live in,” “I have a clear emigration plan,” and “I am fulfilling my emigration plan step by step.”

Students were invited to participate through an informal network of psychologists during regular lectures and by e-mail. Participation was voluntary and anonymous. After the informed consent was received, students filled in the inventory without a time limit.

The *a priori* sample size was calculated using an online calculator (Statistics Calculator, RRID:SCR_013827). Accounting for the anticipated minimal effect size of 0.30, eight observed indicators, two latent variables, alpha level of 0.05, and power of 0.80 resulted in the minimal calculated sample size of 100 for a group. The whole sample and subsamples presenting students’ demographic characteristics (gender, employment, graduation, and income) satisfied this requirement.

Regular statistical tests were performed using IBM SPSS Statistics for Windows 22.0 (IBM SPSS Statistics, RRID:SCR_019096). Confirmatory factor analysis was conducted using “lavaan” 0.6-6 (Rosseel, 2012) for R (R Project for Statistical Computing, RRID:SCR_001905). Model invariance tests were performed using “equaltestMI” 0.6.0 (Jiang et al., 2017) for R (R Project for Statistical Computing, RRID:SCR_001905). Testing invariance focused on the weak (metric), strong (scalar), and strict (residual) equivalence of the model across subgroups (e.g., Putnick and Bornstein, 2016).

## Results

**FIGURE 1:**
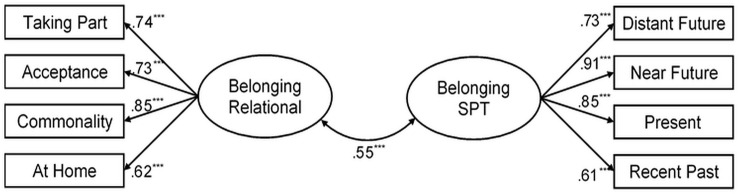
Standardized factor loadings and covariance between the relational and spatiotemporal (SPT) factors of the sense of belonging to the country. ***p < 0.001.

Table 1 presents the results of confirmatory factor analysis testing the suggested two-factor model of belonging to the country and two alternative models—one-factor model and a higher-level factor for both components of the sense of belonging. The Satorra–Bentler correction was applied in the assessment of model fit. The analysis revealed an acceptable fit of Model 2, which corresponded to the model under consideration (Figure 1).

**TABLE 1:** Confirmatory factor analysis of the model of the sense of belonging to the country (*N* = 500).

Model	χ^2^	*df*	CFI	TLI	RMSEA	SRMR
Model 1 (One factor)	400.41	20	0.715	0.602	0.195	0.113
Model 2 (Two factors)	53.58	19	0.974	0.962	0.060	0.037
Model 3 (Higher-order factor)	62.28	18	0.967	0.948	NA	0.037

*NA, not assessed. Model 3 refers to a not well identified model (no standard errors calculated).*

Model 2 was metric invariant, Δχ^2^(6) = 7.09, *p* = 0.311, scalar invariant, Δχ^2^(6) = 4.02, *p* = 0.675, and residual invariant, Δχ^2^(8) = 11.42, *p* = 0.179, regarding students’ gender. Regarding students’ employment, it was also metric invariant, Δχ^2^(6) = 3.87, *p* = 0.694, scalar invariant, Δχ^2^(6) = 2.07, *p* = 0.913, and residual invariant, Δχ^2^(8) = 14.55, *p* = 0.068. Regarding students’ income, it was metric invariant, Δχ^2^(6) = 2.91, *p* = 0.821, scalar invariant, Δχ^2^(6) = 3.22, *p* = 0.780, and residual invariant, Δχ^2^(8) = 13.67, *p* = 0.091. In addition, the model was metric-invariant considering their graduation, Δχ^2^(6) = 6.15, *p* = 0.406, marriage, Δχ^2^(6) = 2.17, *p* = 0.904, and the ethnolinguistic group (comparing Latvian and Russian speakers), Δχ^2^(6) = 4.01, *p* = 0.675.

Table 2 presents the descriptive statistics and intercorrelations among factors of the sense of belonging, demographic variables, and considering emigration. Cronbach’s alpha and test–retest reliability coefficients confirmed acceptable reliability of scales. Both components of the sense of belonging correlated positively with an item presenting the sense of belonging to Latvia explicitly and negatively with considering emigration. The relational component of belonging was more closely associated with the one-item measure, while the spatiotemporal component was more closely associated with considering emigration. Among demographic variables, the ethnolinguistic group and age demonstrated the closest correlations with belonging and considering emigration.

**TABLE 2:** Descriptive statistics, reliability, and intercorrelations of measures of the sense of belonging to the country with considering emigration and demographics (*N* = 500).

Variable	1	2	3	4	5	*M*	*SD*	Items	α	*r*_*xx*_
(1) Relational belonging	–					4.28	1.33	4	0.82	0.77***
(2) Spatiotemporal belonging	0.50***	–				5.71	1.28	4	0.85	0.81***
(3) Belonging to Latvia	0.78***	0.53***	–			5.09	1.73	1	–	0.44**
(4) Considering emigration	−0.27***	−0.54***	−0.29***	–		1.86	1.33	4	0.91	–
(5) Age, years	0.08	0.16***	0.11*	−0.09*	–					
(6) Gender (females)	0.03	0.08	0.04	0.01	0.07					
(7) Working	0.02	0.09	0.00	0.03	0.33***					
(8) Married	0.04	0.10*	0.01	0.08	0.60***					
(9) Median income (higher)	0.08	0.10*	0.06	0.05	0.23***					
(10) Graduated	0.00	0.11*	0.01	0.06	0.65***					
(11) Latvian speakers^*a*^	0.19***	0.19***	0.21***	−0.13**	0.06					

Correlations with the ethnolinguistic group were calculated by comparing Latvian speakers with Russian speakers only; *r*_*xx*_ = test–retest reliability (*n* = 39). ^*a*^*n* = 492. *p < 0.05. **p < 0.01. ***p < 0.001.

It should be noted that temporal categories of the spatiotemporal component demonstrated mutual linear relationships (*R*^2^ varied from 0.19 to 0.61) and suitability for factorization. Simultaneously, their means followed the inverted quadratic trend, *F*(1,499) = 160,90, *p* < 0.001, η^2^ = 0.24, Δη^2^ = 0.03, compared with the linear one. Starting with the past (*M* = 5.86, *SD* = 1.51), it reached the maximum in the present (*M* = 6.15, *SD* = 1.33), followed by the near future (*M* = 5.82, *SD* = 1.47) and the minimum in the distant future (*M* = 5.00, *SD* = 1.82). All pairs but the past and near future demonstrated significant differences.

**FIGURE 2:**
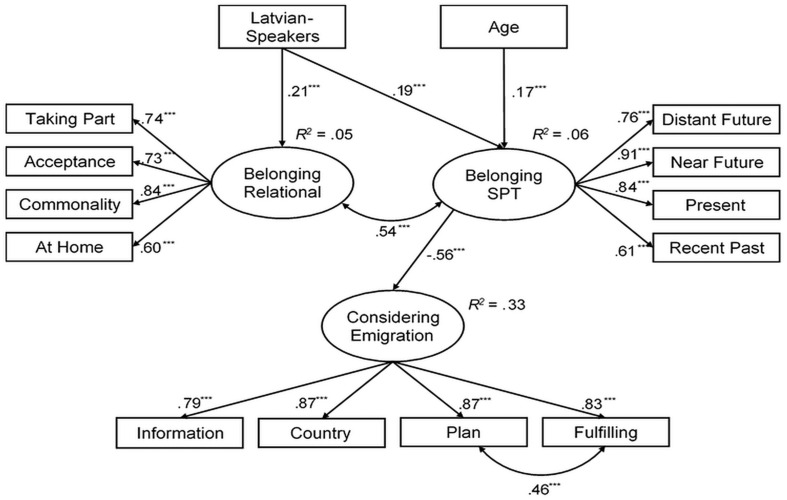
Relational and spatiotemporal (SPT) factors of the sense of belonging to the country and demographic indicators predicting university students’ consideration of emigration. ***p < 0.001.

The predictive role of the sense of belonging to the country in considering emigration was explored by SEM (Figure 2). The model included both components of belonging, students’ age, and the ethnolinguistic group as predictors. The analysis involved 492 university students who identified themselves as Latvian or Russian speakers.

After adding an error covariance between an emigration plan and its implementation, the model demonstrated an acceptable fit: χ^2^(68) = 228.68, *p* < 0.001, CFI = 0.936, TLI = 0.915, RMSEA = 0.069, SRMR = 0.053. A higher level of spatiotemporal belonging was a negative predictor of considering emigration. The relational component of belonging and demographic variables demonstrated no direct effect on it. However, older students were higher on spatiotemporal belonging, Latvian speakers were higher on both components of the sense of belonging, and the relational component of belonging covaried with the spatiotemporal one.

## Discussion

Testing the two-factor model of the sense of belonging to the country confirmed its complex structure. The relational component included the bidirectional process of interaction with a social system at the national level. It affirms the principle of personal involvement and integration into a system, formulated by Hagerty et al. (1992), as the core element of the sense of belonging. The experience of integration also included senses of acceptance, commonality, and being “at home,” mentioned in theoretical analyses and empirical studies (Cameron, 2004; Anthias, 2011; Banting and Soroka, 2012; Berry and Hou, 2016).

The spatiotemporal component integrated personal commitment to the country in a broad range of temporal frames—from the recent past to the distant future. It emphasized the significance of a temporal dimension of identity and belonging, forming their continuity in relation to a social group or system (e.g., Sani et al., 2008; David and Bar-Tal, 2009). It also confirmed theoretical considerations (Miller, 2003; Anthias, 2011) and extended empirical findings on prospective commitment and belonging (Dekel and Tuval-Mashiach, 2012; Kolesovs, 2019) to its retrospective part, previously investigated in a broader context of national identity (Shin et al., 2014).

Identification of two components of the sense of belonging to the country supported the findings of Dekel and Tuval-Mashiach (2012) and indicated that these components are identifiable regardless of traumatic events. Simultaneously, this study does not support the joining of two components of the sense of belonging into one factor or scale (e.g., Dekel and Nuttman-Shwartz, 2009) and demonstrates that one-item measurement is limited in its stability.

The differentiation of relational and spatiotemporal components of the sense of belonging to the country concurs with the view of Anthias (2011) on relative independence of belonging and identification (e.g., self-categorization). Therefore, there is no guarantee for a long-term commitment in the case of a relatively high sense of current integration in the society or low spatiotemporal commitment in the case of low perceived belonging to the country.

Exploring the role of the sense of belonging to the country in considering emigration revealed the different contributions of two factors. The spatiotemporal component was the direct predictor of students’ preparation for emigration. The effect of the relational component is expressed in its interaction with the spatiotemporal one. It emphasizes the role of continuity of identity (Sani et al., 2008; David and Bar-Tal, 2009) and belonging, including its projection into the future (Dekel and Tuval-Mashiach, 2012; Kolesovs, 2019), in understanding emigration intentions and behavior. This result quantifies the contribution of the sense of belonging to considering emigration (Holmes and Burrows, 2012).

The predictive effect of demographic variables was relatively low. Forming the majority in Latvia, Latvian speakers reported a higher sense of relational and spatiotemporal belonging. According to Cameron (2004), observed differences in belonging is a sign of distancing from the country in Russian speakers. It was also observed in the study on prospective belonging to Latvia (Kolesovs, 2019) when Russian speakers demonstrated a lower sense of belonging to the country. Simultaneously, they reported a higher level of belonging to the local community than Latvian speakers. This effect can be re-assessed by applying the current model at both levels.

Students’ age predicted a higher level of spatiotemporal belonging. The correlational analysis shows that age is associated with education, marriage, employment, and income. It presents a complex process of rooting in society. However, the number of participants limited the assessment of relatively small effects, which should be performed in a broader sample.

Another limitation of the study is a relatively homogeneous sample. It was defined by a social problem—the high rate of emigration intentions in students. In further studies, a more representative sample can better reflect tendencies in the general population, while focusing on secondary school students can reveal the development of belonging at an earlier stage. One more limitation addresses the inclusion of the recent past in assessing commitment without a deep insight into personal experiences and historical narratives. This insight forms another issue for further exploration. In addition, the observed decrease of belonging in the distant future indicates that the dynamics of the sense of belonging in the temporal frame can also be explored in depth by investigating possible differences in its trajectories.

In summary, the results confirmed the complexity of the sense of belonging to the country. Integrative bidirectional relationships reflect personal involvement and perceived acceptance, and commitment to the country is expressed in a broad temporal frame—from the recent past to the distant future. The predictive effect of the spatiotemporal component emphasized the role of continuity of belonging in understanding emigration intentions. This finding confirms functional differences between the two dimensions of the sense of belonging.

## Data Availability Statement

The original contributions presented in the study are included in the article/supplementary material, further inquiries can be directed to the corresponding author.

## Ethics Statement

The studies involving human participants were reviewed and approved by Research Ethics Committee of the Institute of Cardiology and Regenerative Medicine of the University of Latvia No 125/2020. Written informed consent for participation was not required for this study in accordance with the national legislation and the institutional requirements.

## Author Contributions

The author confirms being the sole contributor of this work and has approved it for publication.

## Conflict of Interest

The author declares that the research was conducted in the absence of any commercial or financial relationships that could be construed as a potential conflict of interest.
